# Longitudinal changes in children with autism spectrum disorder receiving applied behavior analysis or early start denver model interventions over six months

**DOI:** 10.3389/fped.2025.1546001

**Published:** 2025-05-14

**Authors:** Yusang Du, Xiaozhen Yang, MengMeng Wang, QianQian Lv, Hui Zhou, Gao Sang

**Affiliations:** ^1^ Department of Psychology and Behavioral Sciences, Zhejiang University, Hangzhou, Zhejiang, China; ^2^ Department of Pediatrics, Liaocheng Second People’s Hospital, Liaocheng, Shandong, China; ^3^ Department of Traditional Chinese Medicine, Hangzhou Children’s Hospital, Hangzhou, Zhejiang, China; ^4^ The State Key Lab of Brain-Machine Intelligence, Zhejiang University, Hangzhou, Zhejiang, China

**Keywords:** autism spectrum disorder, intervention training, Applied Behavior Analysis Therapy, early start denver model, rehabilitation autism spectrum disorder, rehabilitation

## Abstract

**Background:**

Autism Spectrum Disorder (ASD) is a complex neurodevelopmental disorder characterized by social communication difficulties, restricted interests, repetitive behaviors, and sensory abnormalities. The rising prevalence of ASD presents a significant public health concern, with no pharmacological treatments available for its core symptoms. Therefore, early and effective behavioral interventions are crucial to improving developmental outcomes for children with ASD. Current interventions primarily focus on educational rehabilitation methods, including Applied behavior Analysis (ABA) and the Early Start Denver Model (ESDM).

**Objective:**

This study aims to examine the developmental changes in children with ASD following six months of ABA therapy or ESDM intervention.

**Methods:**

From December 2021 to December 2023, 30 children receiving ABA therapy at the Zhejiang Rehabilitation Medical Center (40 min/session, 4 sessions/day, 5 days/week), while another 30 children undergoing ESDM training at Hangzhou Children's Hospital (2 h of one-on-one sessions and 0.5 h of group sessions/day, 5 days/week). Both groups participated in their respective interventions for six months. Pre- and post-treatment assessments were conducted using the Psycho-educational Profile-Third Edition (PEP-3).

**Results:**

Both groups showed significant improvements in PEP-3 scores post-treatment, including cognitive verbal/pre-verbal, expressive language, receptive language, social reciprocity, small muscles, imitation, emotional expression, and verbal and nonverbal behavioral characteristics.

**Conclusion:**

Both ABA and ESDM interventions were associated with comprehensive improvements in children with ASD over a six-month period.

## Introduction

Autism Spectrum Disorder (ASD), also known as autism, is a complex neurodevelopmental condition characterized by impairments in social communication, restricted interests, repetitive behaviors, and sensory abnormalities (1). In recent years, the prevalence of ASD has been steadily increasing worldwide. In 2020, it was estimated that among children aged 8 years, one in 36 was diagnosed with ASD. Specifically, approximately 4% of boys and 1% of girls in this age group were affected (2). In China, a national multi-center study conducted in 2020 found a prevalence of 0.7% among children aged 6 to 12, with 68.8% of children with ASD having at least one neuropsychiatric comorbidity (3). The multifactorial nature of ASD arises from the dynamic interplay of genetic predispositions and environmental factors, highlighting the importance of early identification and timely intervention to optimize developmental outcomes (4). Early childhood development is a critical period in the etiology of ASD, during which disruptions such as zinc deficiencies impacting synaptic development (5), epigenetic changes associated with advanced paternal age (6), maternal immune activation, and exposure to environmental toxicants during pregnancy (7, 8) can significantly affect neurodevelopment. These findings underscore the complex and interconnected biological and environmental processes that contribute to ASD.

Despite the increasing prevalence and growing awareness of immense burden caused by ASD, there are no specific biomarkers for ASD diagnosis, and its etiology remains unclear (9–11). The symptoms of ASD can persist throughout life and may significantly impact individuals' ability to live independently or perform daily activities (12). Given the lack of pharmacological treatments targeting the core symptoms of ASD, behavioral interventions play a critical role in addressing developmental delays, improving social communication, and enhancing adaptive skills (9, 10). Early intervention is particularly important, as it capitalizes on the neuroplasticity of young children, offering the greatest potential for long-term positive outcomes. Especially when combined with rehabilitation training and occasionally medication, early intervention can significantly alleviate symptoms and reduce the risk of further complications (10).

Applied Behavior Analysis (ABA) and The Early Start Denver Model (ESDM) interventions are widely recognized and evidence-based interventions for ASD. ABA, also known as behavior therapy or behavior modification, employs a “stimulus-response-reinforcement” model (11, 13). It breaks down skills into small steps for repetitive practice, with timely rewards and punishments based on principles of reinforcement, extinction, punishment, and shaping. Comprehensive and intensive ABA training has been shown to improve learning, logic, communication, and adaptive skills in children with ASD (14). ESDM was developed by Rogers and Dawson and introduced to China by Professor Xu Xiu of Fudan University's Pediatric Hospital in 2013 (15, 16). ESDM is a comprehensive early intervention model grounded in developmental science, centered on the children's interests, and adaptable to various settings (15). It emphasizes parent-child interaction, using play to elicit positive emotions and promote skill development (15, 17). While both ABA and ESDM have demonstrated effectiveness in improving core symptoms of ASD in controlled settings (18), significant gaps remain in understanding their real-world implementation. First, while long-term outcome studies exist (more than one year), they often overlook critical developmental transitions occurring during the 6-month intervention window (18, 19). Second, available short-term data frequently lack comprehensive assessments of symptom evolution (20). Third, systematic reporting of interim outcomes (3–6 months) in ABA interventions remains scarce, with fewer than 20% of studies documenting progress at this clinically relevant timeframe (21). The current study addresses these limitations through longitudinal assessment of developmental changes across all PEP-3 subscales following six months of intervention, providing much-needed empirical data to inform clinical practice during this pivotal treatment period.

In this study, we have chosen to use person-first language rather than identity-first language. This decision aligns with clinical and research conventions, which often prioritize person-first language to emphasize the individual rather than the condition. Person-first language is widely used in medical and psychological literature to promote respect and reduce stigma by recognizing the person as distinct from their diagnosis (22, 23). However, we acknowledge the ongoing debate within the autism community regarding language preferences. Many self-advocates and autistic individuals prefer identity-first language as it reflects the view that autism is an integral part of their identity rather than a separate condition (24). While we respect and value these perspectives, our choice of person-first language is intended to align with the conventions of the broader scientific and clinical audience while remaining sensitive to the diversity of preferences within the autism community.

It is hypothesized that both ABA and ESDM are effective in improving the core symptoms of ASD. This study evaluates the efficacy of ABA and ESDM in improving core ASD symptoms over six months, using Psycho-educational Profile-Third Edition (PEP-3) as a comprehensive outcome measure. By exploring the value of these interventions, the study intends to provide valuable insights for the promotion of suitable rehabilitation training models for ASD children.

## Participants and methods

### Participants

A total of 60 ASD children were recruited for this study. Thirty children were recruited from Zhejiang Rehabilitation Medical Center from December 2021 to December 2023, and received rehabilitation training using Applied Behavior Analysis Therapy (ABA group). Other 30 children were recruited from Hangzhou Children's Hospital during the same period, and all these children underwent rehabilitation training using the Early Start Denver Model (ESDM group). The following inclusion and exclusion criteria were used, no participants were excluded from this study. The specific criteria are outlined as follows.

The program was approved by the Research Ethics Committee of the hospitals (Zhejiang Rehabilitation Medical Center, Identifier: 2020-LY-098. Hangzhou Children's Hospital, Identifier: 2020-CR-06). Parents or other legal guardians of the child were fully informed and provided consent to participate in the study.

### Diagnostic criteria

The clinical diagnosis of ASD was based on the American Psychiatric Association's Diagnostic and Statistical Manual of Mental Disorders, Fifth Edition (DSM-5) (1), and the Expert Consensus on Early Diagnosis of Autism Spectrum Disorder in Young Children published by the Chinese Medical Association's Pediatrics Branch in July 2022 (3).

### Inclusion criteria

Children diagnosed with ASD who also met the diagnostic criteria of the Autism Diagnostic Observation Schedule-Second Edition (ADOS-2) were included (25). Specifically, the diagnostic criteria were that the scores across all four dimensions of the ADOS-2 exceeded the established ASD cutoff points. These cutoff points were used to identify ASD severity across different dimensions. Higher scores on these dimensions indicate greater severity of autism-related symptoms. The cutoff points for each dimension are as follows:

Dimension 1 (communication): Autism ≥ 12
Dimension 2 (social interaction): Autism ≥ 10
Dimension 3 (playing): Autism ≥ 9
Dimension 4 (restricted and repetitive behaviors): Autism ≥ 10

### Exclusion criteria

Children met any of the following criteria were excluded from the study: (1) experiencing severe illness during the investigation that could affect the assessment of efficacy, such as epilepsy, advanced cancer or severe depression; (2) diagnosed with comorbid conditions (e.g., hearing impairment, global developmental delay, isolated speech delay, intellectual disability, psychiatric disorders, or genetic metabolic diseases) during the study, which would interfere with the corresponding intervention treatment; (3) receiving concurrent interventions during the study that could affect the evaluation of efficacy.

### Elimination criteria

Children who met any of the following criteria were eliminated from the study: (1) unwillingness to continue participation or withdrawal during the investigation; (2) failure to cooperate with data collectors in adhering to the established data collection procedures.

### Study procedure

Prior to intervention and rehabilitation treatment, physicians specializing in child development and behavior conducted assessments of the children's behavioral characteristics, cognitive levels, growth and development levels, and collected descriptions of daily behavior from their families. To ensure consistency across the study, all personnel received uniform training on diagnostic criteria, and intervention methods.

Both groups participated in a continuous rehabilitation intervention for six months. The Psycho-educational Profile-Third Edition (PEP-3) was conducted to evaluate participants' skills and behaviors both before training and after a six-month training period, enabling a comprehensive analysis of the training program's effectiveness (26). The details of PEP-3 were listed on the *Measures* part.

### Intervention procedure

ABA Group: The training focused on improving speech, imitation, language, daily living skills, social skills, gross motor skills, fine motor skills, and cognitive abilities. The training included activities such as pronunciation, picture recognition, language action imitation, dressing, simple to complex games, roller training, and puzzles. The training process emphasized breaking down skills into behavior units and reinforcing them until they could be combined into complex behaviors. Participants received 4 ABA training sessions per day (5 days a week), with each session lasting 40 min. The whole intervention lasting for 6 months.

ESDM Group: Before the intervention, therapists put effort on communicating with the children and their families, developed individualized teaching plans based on the children's interests, and focused on enhancing abilities in language comprehension and expression, social cognition, gross motor skills, fine motor skills, and imitation through play. Parents accompanied their children throughout the intervention and were encouraged to participate. The training included one-on-one sessions focusing on language and cognitive training for 2 h daily and group sessions focusing on motor skills and social rule awareness for 0.5 h daily, 5 days a week. The whole intervention lasting for 6 months.

### Measures

The Chinese version of the Psycho-educational Profile-Third Edition (PEP-3), was used to evaluate the developmental abilities and behavioral characteristics of children before and after intervention. It was used to assess the behavioral characteristics of children with ASD from preschool to elementary school age, based on standardized observation of their performance, which focuses on individualized evaluation of developmental skills, strengths, deficits, and behaviors. The Chinese version of the PEP-3 demonstrates strong psychometric properties, including high internal consistency (α = 0.89–0.97), test-retest reliability (0.84–0.99), and inter-rater reliability (0.34–0.87) (27). Its validity is supported by item analyses and significant correlations with the Merrill-Palmer Revised Scales of Development (MPR) and the Childhood Autism Rating Scale (CARS) (27). These scores could provide insights into a child's strengths and weaknesses, guiding individualized educational and behavioral intervention plans. The PEP-3 consists of 10 sub-scales. Among them, three sub-scales assess communication skills: cognitive verbal/pre-verbal (CVP) (34 items), expressive language (EL) (25 items), and receptive language (RL) (19 items). Another three sub-scales evaluate motor abilities: fine motor (FM) (20 items), gross motor (GM) (15 items), and visual motor imitation (VMI) (10 items). These six sub-scales focus on assessing a child's developmental level. The remaining four sub-scales measure maladaptive behaviors, including affective expression (AE) (11 items), social reciprocity (SR) (12 items), characteristic motor behavior (CMB) (15 items), and characteristic verbal behavior (CVB) (11 items). These sub-scales are used to assesses emotional responsiveness, reciprocal social interactions, repetitive or stereotyped movements, and atypical speech patterns, all of which are core features of ASD. The structured scoring system, ranging from “Pass” (2 points) to “Emerge” (1 point) and “Fail” (0 points), enables professionals to systematically assess and compare developmental progress across different groups and over time (27). Higher scores indicate better abilities in the assessed dimensions (26).

### Statistical analysis

All data were entered using Excel software and statistically analyzed using SPSS (https://www.ibm.com/spss) and JASP software (https://jasp-stats.org/). Given the non-randomized allocation of participants into the two intervention groups during recruitment, our primary analytical focus centered on within-group treatment effects to mitigate design limitations.

First, we used Shapiro–Wilk test to evaluate the normality of the data distribution (Supplementary Material S1). For the data that conforms to the normal distribution, we used paired *t*-tests to examine the differences between pre- and post- intervention in two groups; non-normally distributed measurement data were analyzed using Wilcoxon signed-rank test. Mean value and standard deviation ($\bar{X}\pm S$) were used to describe the data.

As exploratory analyses, between-group comparisons were conducted using linear mixed models with intervention type (ABA vs. ESDM) and time (pre- vs. post-intervention) as fixed factors, and participant as a random intercept factor to account for repeated measurements. We are fully acknowledged of potential confounding from group allocation that may influence between-group interpretations. Results from exploratory analyses are reported with this limitation in mind. Gender comparisons were analyzed using Chi-square tests. Age comparisons were analyzed using Mann–Whitney *U*-tests. Effect sizes were represented by Rank-Biserial Correlation (r_rb) or Cohen's d (d) to accommodate parametric and non-parametric data distributions. The False Discovery Rate (FDR) correction was applied to adjust for multiple comparisons and control the risk of Type I errors. A corrected *p*-value of less than 0.05 was considered statistically significant.

## Results

### Comparison of PEP-3 scores before and after 6 months of intervention treatment in ABA group

After 6 months of treatment, the PEP-3 scores of the ABA group showed significant improvement in all 10 sub-scales compared to that before the treatment, with differences being statistically significant (all FDR Adjusted *p* *<* 0.05, Table 1).

**Table 1:** Comparison of PEP-3 scores before and after 6 months of intervention in the ABA group.

Sub-scales	pre-intervention	post-intervention	Z(T)-value	r_rb(d)-value	*P*-value	FDR Adjusted *P*-value
Cognitive verbal/pre-verbal (CVP)	32.733 ± 17.064	41.2 ± 14.938	3.911(T)	0.714(d)	<0.001***	<0.001***
Expressive language (EL)	15.533 ± 12.108	21.867 ± 12.588	3.969	0.908	<0.001***	<0.001***
Receptive language (RL)	21.067 ± 10.514	26.533 ± 9.024	3.448	0.759	<0.001***	<0.001***
Fine motor (FM)	26.933 ± 9.417	32.233 ± 5.87	4.190	0.906	<0.001***	<0.001***
Gross motor (GM)	21.2 ± 7.49	25.3 ± 5.453	3.543	0.844	<0.001***	<0.001***
Visual motor imitation (VMI)	12.667 ± 5.542	15.367 ± 4.198	3.5	0.817	<0.001***	<0.001***
Affective expression (AE)	7.833 ± 4.348	9.733 ± 5.527	2.658 (T)	0.485 (d)	<0.001***	<0.001***
Social reciprocity (SR)	9.733 ± 4.66	11.9 ± 5.827	3.295 (T)	0.602 (d)	<0.001***	<0.001***
Characteristic motor behavior (CMB)	15.533 ± 6.112	18.4 ± 6.642	3.165	0.771	0.002***	0.002***
Characteristic verbal behavior (CVB)	6 ± 4.323	9 ± 4.906	3.416	0.871	<0.001***	<0.001***

***p ≤ 0.001; Z-values are reported for non-parametric tests as standardized test statistics, T-values are used as test statistics in parametric tests.

### Comparison of PEP-3 scores before and after 6 months of intervention treatment in ESDM group

After 6 months of treatment, the PEP-3 scores of the ESDM group showed significant improvement in all 10 sub-scales compared to that before the treatment, with differences being statistically significant (all FDR Adjusted *p* *<* 0.05, Table 2).

**Table 2:** Comparison of PEP-3 scores before and after 6 months of intervention in the ESDM group.

Sub-scales	pre-intervention	post-intervention	Z(T)-value	r_rb(d)-value	*P*-value	FDR Adjusted *P*-value
Cognitive verbal/pre-verbal (CVP)	38.833 ± 16.329	49.833 ± 12.231	10.375 (T)	1.894(d)	<0.001***	<0.001***
Expressive language (EL)	18.9 ± 12.604	27.967 ± 11.14	4.782	1.000	<0.001***	<0.001***
Receptive language (RL)	22 ± 11.471	30.433 ± 7.623	4.782	1.000	<0.001***	<0.001***
Fine motor (FM)	31.9 ± 6.905	36.167 ± 4.34	4.457	1.000	<0.001***	<0.001***
Gross motor (GM)	27.433 ± 4.695	28.933 ± 2.132	3.408	1.000	<0.001***	<0.001***
Visual motor imitation (VMI)	14.6 ± 4.304	17.7 ± 2.996	4.541	1.000	<0.001***	<0.001***
Affective expression (AE)	14.967 ± 3.113	16.867 ± 2.240	6.238 (T)	1.139 (d)	<0.001***	<0.001***
Social reciprocity (SR)	13.367 ± 4.460	17.567 ± 3.757	11.366 (T)	2.075 (d)	<0.001***	<0.001***
Characteristic motor behavior (CMB)	22.367 ± 5.176	24.8 ± 3.448	4.286	1.000	<0.001***	<0.001***
Characteristic verbal behavior (CVB)	9.767 ± 6.361	11.9 ± 7.303	4.107	1.000	<0.001***	<0.001***

***p ≤ 0.001; Z-values are reported for non-parametric tests as standardized test statistics, T-values are used as test statistics in parametric tests.

### Comparison of general information and pre-intervention scores between the two groups

The Chi-square test indicated no significant gender difference between the two groups (*χ*^2^ = 0.373, *p* = 0.542, Table 3). In addition, no significant age difference between the two groups was identified using Mann–Whitney *U*-tests (*Z* = 0.015, *p* = 0.988, Table 3).

**Table 3:** Comparison of gender and Age between the Two groups.

Group	Sample size	Male (*n*, %)	Female (*n*, %)	Chi-square	*P*-value	Age	Z-value	*P*-value
ABA group	30	24 (80%)	6 (20%)	0.373	0.542	4.53 ± 1.45	0.015	0.988
ESDM group	30	22 (73.3%)	8 (26.7%)			4.58 ± 1.58		

### Comparison of PEP-3 scores before and after 6 months of intervention treatment between the two groups

**Figure 1:**
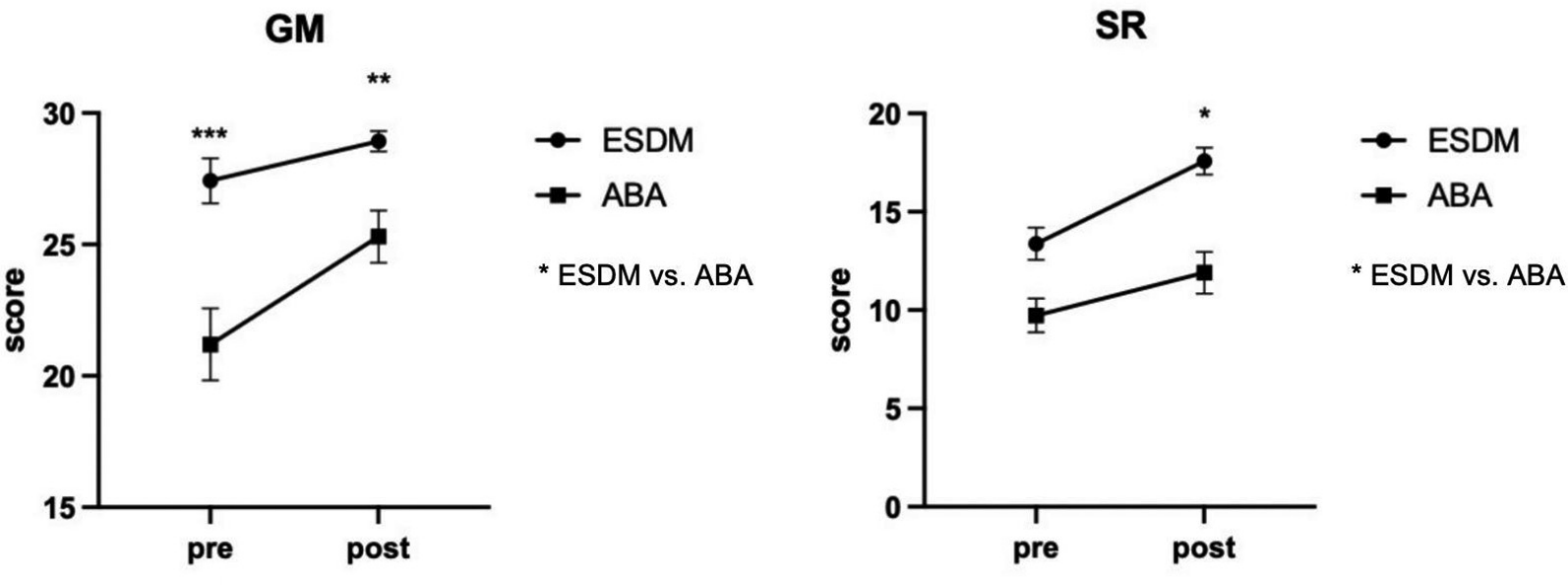
Estimated marginal means ± SEM for GM and SR domains showing significant group × time interaction effects (*p* < 0.05). * indicates significant between-group pre- and post- intervention differences (*p *<* 0.05, **p ≤ 0.01, ***p ≤ 0.001).

Between-group comparisons were conducted as exploratory analyses using linear mixed models. After 6 months of intervention, the ESDM group showed greater improvement compared to the ABA group in scores for SR [F (1,58) = 7.27, *p_uncorrected_* = 0.009, Table 4, Figure 1], while ABA group shows greater improvement in GM [F (1,58) = 4.74, *p_uncorrected_* = 0.034, Table 4, Figure 1] comparing to ESDM group. However, these between-group differences were no longer statistically significant after FDR correction.

**Table 4:** Linear mixed model results for intervention and time effects on PEP-3 scores for two groups.

Intervention	F-value	Cohen's d	*P*-value	FDR Adjusted *P*-value
cognitive verbal/pre-verbal (CVP)	1.104	0.680	0.298	0.423
expressive language (EL)	2.935	0.840	0.092	0.230
receptive language (RL)	2.867	0.910	0.096	0.230
fine motor (FM)	0.591	−0.340	0.445	0.636
gross motor (GM)	4.742	0.130	0.034*	0.170
visual motor imitation (VMI)	0.245	−0.140	0.623	0.779
affective expression (AE)	0.000	0.000	1.000	1.000
social reciprocity (SR)	7.267	0.700	0.009**	0.090
characteristic motor behavior (CMB)	0.227	0.990	0.636	0.779
characteristic verbal behavior (CVB)	1.112	−0.290	0.296	0.423

* *p* < 0.05, **p ≤ 0.01. Linear mixed models were fitted with intervention type (ABA vs. ESDM) and time (pre- vs. post-intervention) as fixed factors, and participant as a random intercept factor to account for repeated measurements.

## Discussion

Our findings revealed that both ABA and ESDM interventions effectively improved various abilities in children with ASD as measured by the PEP-3. These results suggest that both ABA and ESDM methods are effective in enhancing the overall quality of life for children with ASD, enabling them to better adapt to their daily environments. The observed improvements in these abilities and skills hold great clinical significance, as they reflect substantial functional gains that are highly relevant and applicable in real-world settings: Enhanced cognitive-verbal and language skills (CVP/EL/RL) enable better self-expression and social understanding, refined motor abilities (FM/GM/VMI) support daily functioning and play participation, while reduced challenging behaviors (AE/SR/CMB/CVB) facilitate more adaptive social interactions and learning engagement–collectively promoting meaningful integration into home, school, and community settings. These improvements collectively contribute to a higher quality of life and greater independence for individuals with ASD.

ASD is a neurodevelopmental disorder characterized by considerable heterogeneity in symptoms (17). Core challenges in children with ASD often include reduced behaviors such as looking, pointing, responding, speaking, and appropriate social interaction, alongside comorbid conditions like ADHD, anxiety, depression, epilepsy, and motor coordination difficulties (28, 29). While some individuals with ASD display exceptional skills in areas such as memory and music, many experience regression in language or social abilities around 18–24 months (9, 29, 30). ASD poses significant challenges for families, with parents often facing heightened stress, anxiety, and financial burdens due to the chronic nature of the condition (31). As individuals with ASD transition into adulthood, health challenges can intensify. Early intervention, particularly through behavioral therapies, has shown promise in improving core symptoms (32).

Our study have demonstrated that both ABA and ESDM methods significanly improved target abilities, which confirmed the effectiveness of both methods. ABA is generally considered an effective intervention method in previous studies (21). For example, the application of “Antecedent, Behavior, and Consequence(ABC)” model, which forms the fundamental of ABA, resulted in 47% of children undergoing this treatment achieving normal intellectual and educational functioning (21, 33, 34). A meta-analysis of the effects of ABA intervention on ASD, including 14 randomized controlled trials with 555 participants, revealed that ABA intervention improved social communication and language expression (35). Whereas another meta-ananlysis reported moderate effects on intellectual functioning and adaptive behavior in children with ASD (35). A retrospective observational case study demonstrated that after 12 months of ABA-based intervention, children were able to independently perform most of the proposed tasks, particularly those related to academic, social, and daily living skills (36). Notably, even one month of intervention training could significantly improve the target behaviors (34). Our study further supports the effectiveness of this method, showing that after six months of ABA intervention, the target behaviors in children with ASD were significantly improved.

ESDM is a comprehensive early intervention method for children with ASD that integrates applied behavior analysis and developmental science to enhance natural learning opportunities through daily activities, play, and experiences (37–41). It emphasizes parent-child interactions and can be delivered by various professionals across different settings, including clinics, homes, and schools (41, 42). Previous studies have demonstrated the effectiveness of ESDM with varying intervention durations and intensities. For instance, a six-month low-intensity ESDM intervention resulted in significant improvements in language and overall cognitive function, along with reduced symptom severity in communication and play, outperforming routine community treatments (20, 41, 42). Another study implemented ESDM sessions lasting two hours per week for one year, showing great progress in communication, social skills, and reductions in challenging behaviors (37). Additionally, ESDM was found to uniquely influence neural circuits underlying social cognition and familiarity, as evidenced by greater mu rhythm attenuation during a grasping task in participants who underwent ESDM (39). Our findings further confirmed that after six months of intervention, the ESDM group showed significant improvements across all assessed sub-dimensions, including language, social interaction, and motor abilities, highlighting the broad and transformative potential of ESDM in enhancing developmental outcomes for children with ASD.

While the primary focus of this study was to evaluate the overall efficacy of both interventions, exploratory analyses revealed tentative trends suggesting that the ESDM group may demonstrate greater improvement than the ABA group social reciprocity (*p* = 0.009) domains, while ABA group shows greater improvement in gross motor (*p* = 0.034) comparing to ESDM group. However, these interaction effects did not survive FDR correction (GM: *p* = 0.17; SR: *p* = 0.09). Current evidence doesn't support preference for ESDM in GM/SR domains, but offers valuable hypotheses for personalized intervention. The medium effect size in SR (d = 0.70) might reflect ESDM's play-based strategies particularly targeting social communication core deficits, consistent with Rogers' joint attention facilitation hypothesis (15). Future studies could incorporate neurophysiological markers to elucidate underlying mechanisms.

The effectiveness of behavioral therapies has been proved influenced by several factors. For ESDM, studies suggest that younger age (19) and milder baseline symptom severity may enhance responsiveness, though improvements are observed across severity levels with appropriate adaptations. Similarly, ABA outcomes vary based on intervention intensity, cognitive profile, and family engagement (35). Crucially, both approaches demonstrate optimal effects when individualized to the child's developmental needs—ESDM through play-based naturalistic strategies and ABA via structured skill sequencing. This underscores the importance of personalized intervention planning rather than direct protocol comparisons, as both methods can promote significant gains across core ASD domains when properly matched to the child's profile. These effects highlight the importance of tailoring interventions based on individual characteristics, as one approach may not universally outperform another across all skill domains.

This study provides longitudinal evidence that both ABA and ESDM interventions elicit significant improvements across multiple developmental domains in children with ASD, as measured by comprehensive PEP-3 assessments. Importantly, our six-month intervention data suggest that clinical decision-making should prioritize: (1) matching intervention components to the child's specific symptom profile (e.g., greater focus on ESDM for social communication deficits vs. ABA for adaptive behavior needs), and (2) continuous progress monitoring to allow dynamic adaptation of therapeutic approaches. These findings support the emerging practice paradigm of personalized, multidimensional intervention planning in ASD rehabilitation.

While our research contributes valuable insights, it is crucial to acknowledge certain limitations and outline future directions to address them. First, due to the limited sample size, no subgroup analysis was conducted based on the severity of the condition. Future research should focus on larger and more diverse samples, enabling subgroup analyses based on autism severity, age, and other demographic factors to explore differential intervention effects. Second, the study did not account for learning rates or other time-related factors, which may have influenced the interpretation of intervention outcomes. Additional time-point measurements, such as mid-intervention assessments and long-term follow-ups at 3 months, 6 months, or even 1–2 years post-intervention, should be included to analyze learning rates and the sustainability of intervention outcomes. Moreover, variations in the proficiency level of therapists providing rehabilitation interventions may lead to variability in children's responses to ESDM. Other potential influencing factors, such as the intervention location, family income, caregivers' educational level, the duration since the child's ASD diagnosis, caregivers' beliefs about the treatment, and their comfort in accessing services, were not systematically examined. Future research should standardize intervention intensity and format across groups to ensure comparability and investigate the impact of different intervention settings, such as inclusive vs. specialized environments, on outcomes. Finally, the role of family involvement, socioeconomic factors should be systematically explored to better understand their influence on intervention efficacy. By addressing these limitations and pursuing these future directions, researchers can provide a more comprehensive understanding of intervention effectiveness and inform personalized, evidence-based practices for children with autism.

In summary, both ABA and ESDM have certain intervention effects on the rehabilitation of children with ASD in six-months period. From a long-term perspective, it is a reasonable and effective sustained rehabilitation program for both children and their parents.

## Conclusion

Both Applied Behavior Analysis (ABA) therapy and the Early Start Denver Model (ESDM) both significantly improve core ASD symptoms within six months. Clinicians may consider either approach based on child-specific needs and contextual factors, as both demonstrate robust efficacy.

## Data Availability

The raw data supporting the conclusions of this article will be made available by the authors, without undue reservation.
